# Poly[bis­(μ-azido-κ^2^ *N* ^1^:*N* ^1^)[μ-1,2-bis­(imid­azol-1-yl)ethane-κ^2^ *N* ^3^:*N* ^3′^]cadmium]

**DOI:** 10.1107/S1600536812004370

**Published:** 2012-02-10

**Authors:** Hai-Yan Li, Peng-Peng Sun, Bao-Long Li

**Affiliations:** a College of Chemistry, Chemical Engineering and Materials Science, Key Laboratory of Organic Synthesis of Jiangsu Province, Soochow University, Suzhou 215123, People’s Republic of China

## Abstract

In the title three-dimensional coordination polymer, [Cd(N_3_)_2_(C_8_H_10_N_4_)]_*n*_, the coordination geometry around the Cd^II^ atom is distorted octa­hedral. The Cd^II^ atom is coordinated by two N atoms from two *cis*-positioned bridging 1,2-bis­(imidazol-1-yl)ethane (bime) ligands and four N atoms from four azide anions. Each azide ligand acts in an end-on bridging coordination mode. The azide ligands and Cd^II^ atoms form a one-dimensional zigzag chain constructed from four-membered [Cd(N_3_)_2_]_*n*_ metallacycles extending along the *a* axis. These inorganic chains are connected with four other chains *via* bridging bime ligands to form a three-dimensional coordination network.

## Related literature

For coordination polymers with intriguing structures, see: Batten & Robson (1998 ▶); Blake *et al.* (1999 ▶); Kitagawa *et al.* (2004 ▶). For coordination polymers with flexible ligands, see: Hoskins *et al.* (1997*a* ▶,*b* ▶). For azide coordination compounds and polymers, see: Ribas *et al.* (1999 ▶); Leibeling *et al.* (2004 ▶); Chen & Chen (2002 ▶); Mautner *et al.* (1997 ▶). For 1,2-bis­(imidazol-1-yl)ethane (bime) coordination polymers, see: Zhang *et al.* (2005 ▶, 2008 ▶); Zhu *et al.* (2010 ▶).
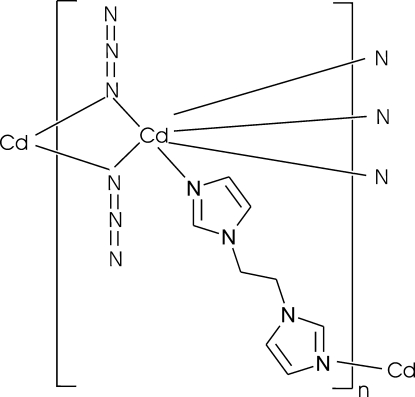


## Experimental

### 

#### Crystal data

[Cd(N_3_)_2_(C_8_H_10_N_4_)]
*M* *_r_* = 358.66
Monoclinic, 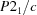

*a* = 6.4565 (14) Å
*b* = 18.874 (4) Å
*c* = 10.449 (2) Å
β = 90.485 (5)°
*V* = 1273.2 (5) Å^3^
*Z* = 4
Mo *K*α radiation
μ = 1.72 mm^−1^
*T* = 153 K
0.36 × 0.17 × 0.15 mm

#### Data collection

Rigaku Mercury CCD diffractometer
Absorption correction: multi-scan (*REQAB*; Jacobson, 1998 ▶) *T*_min_ = 0.576, *T*_max_ = 0.783
12260 measured reflections
2324 independent reflections
2190 reflections with *I* > 2σ(*I*)
*R*_int_ = 0.029

#### Refinement

*R*[*F* ^2^ > 2σ(*F* ^2^)] = 0.023
*wR*(*F* ^2^) = 0.056
*S* = 1.09
2324 reflections
172 parameters
H-atom parameters constrained
Δρ_max_ = 0.68 e Å^−3^
Δρ_min_ = −0.39 e Å^−3^

### 

Data collection: *CrystalClear* (Rigaku, 2000 ▶); cell refinement: *CrystalClear*; data reduction: *CrystalClear*; program(s) used to solve structure: *SHELXS97* (Sheldrick, 2008 ▶); program(s) used to refine structure: *SHELXL97* (Sheldrick, 2008 ▶); molecular graphics: *SHELXTL* (Sheldrick, 2008 ▶); software used to prepare material for publication: *SHELXTL*.

## Supplementary Material

Crystal structure: contains datablock(s) global, I. DOI: 10.1107/S1600536812004370/gk2436sup1.cif

Structure factors: contains datablock(s) I. DOI: 10.1107/S1600536812004370/gk2436Isup2.hkl

Additional supplementary materials:  crystallographic information; 3D view; checkCIF report

## Figures and Tables

**Table:** 

Cd1—N2	2.306 (2)
Cd1—N4^i^	2.324 (2)
Cd1—N5	2.340 (2)
Cd1—N8	2.345 (2)
Cd1—N8^ii^	2.377 (2)
Cd1—N5^iii^	2.397 (2)

**Table:** 

N2—Cd1—N5	91.57 (8)

Symmetry codes: (i) 
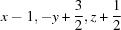
; (ii) 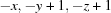; (iii) 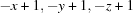.

**Fig. 1.**
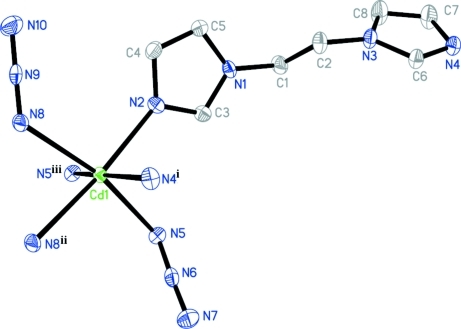
The coordination environment of Cd1 in (I) with displacement ellipsoids drawn at the 50% probability level [symmetry codes: ( i) x - 1, -y + 3/2, z + 1/2; (ii) -x, -y + 1, -z + 1; (iii) -x + 1, -y + 1, -z + 1].

**Fig. 2.**
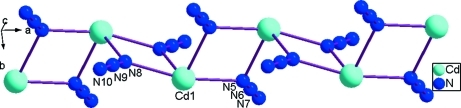
The one-dimensional inorganic chain with bridging EO-azide ligands running along the a axis.

**Fig. 3.**
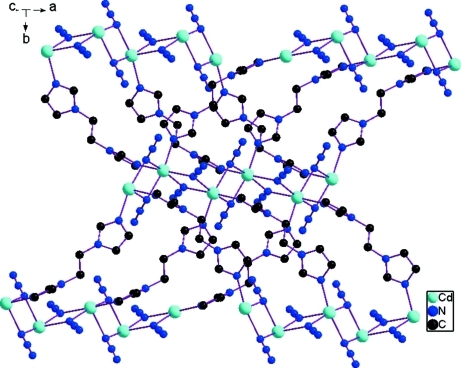
Inorganic [Cd(N3)2]n chains bridged by bime ligands.

**Fig. 4.**
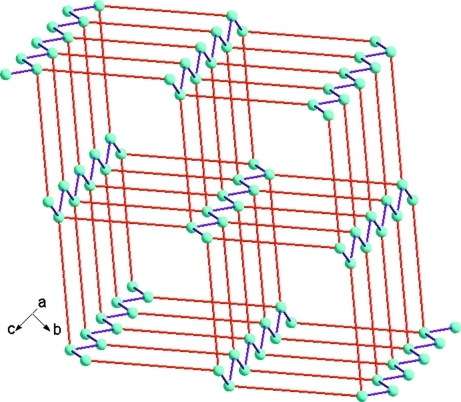
A three-dimensional topology of (I). The long sticks represent the bime ligands and the short ones the pair of EO-azide ligands.

## supplementary crystallographic information

### Comment

Coordination polymers have been paid great attention for their intriguing structures and potential applications as functional materials (Batten & Robson 1998; Kitagawa *et al.*, 2004). The selection of ligand is very important for the design and construction of the coordination polymers. In contrast to the rigid ligands with little or no conformational freedom when they interact with the metal ions, the flexible ligands can adjust their conformations to the geometric requirements of the metal ions (Hoskins *et al.*, 1997**a*,b*).

The short anion ligand azide (N_3_^-^) is widely used to construct novel coordination polymers because its versatile coordination modes and the ability to mediate strong magnetic coupling (Ribas *et al.*, 1999; Leibeling *et al.*, 2004).

In our previous studies, we synthesized two Cd^II^ coordination polymers with the flexible ligand 1,2-bis(imidazol-1-yl)ethane (bime; Zhang *et al.*, 2005; Zhang *et al.*, 2008) and one Cu^II^ coordination polymer (Zhu *et al.*, 2010). In order to extend our work, in the present paper we report the preparation and crystal structure of a novel three-dimensional cadmium(II) coordination polymer [Cd(bime)(N_3_)_2_]_n_ (I) with the flexible ligand bime and short anion ligand azide.

The structure of (I) is a novel three-dimensional network. Each Cd^II^ atom is coordinated by six nitrogen atoms: two from two bime ligands in the *cis*-positions and the remaining four from four azide anions, in a distorted octahedral geometry (Fig. 1, Table 1).

The Cd—N bond lengths are in the range of 2.306 (2) - 2.397 (2) Å and do not vary much. The Cd—N (bime) lengths of 2.306 (2) and 2.324 (2)Å are corresponding to the values 2.2769 (17)Å in [Cd(bime)dca)_2_]_n_ (dca = dicyanamide; Zhang, *et al.*, 2005) and 2.2748 (18) Å in [Cd(phba)_2_(bime)(H_2_O)_2_]_n_ (phba = 4-hydroxybenzoate; Zhang *et al.*, 2008). There are two symmetry independent azide ligands and they both act in end-on (EO) bridging coordination mode. The EO-azide ligands are linear and asymmetric [bond angles N5—N6—N7 = 177.5 (3)° and N8—N9—N10 = 178.4 (3)°, bond lengths N5—N6 = 1.195 (3) Å, N6—N7 = 1.158 (3) Å, N8—N9 = 1.166 (3) Å and N9—N10 = 1.177 (4) Å].

The Cd—N (azide) bond lengths of 2.340 (2) to 2.397 (2)Å are similar to the corresponding values of 2.258 (7) and 2.335 (6) Å in [Cd(picolinato)(N_3_]_n_ with only EO-azide (Mautner *et al.*, 1997) and 2.404 (3) to 2.474 (3) Å in [Cd(N_3_)(4-aba)(H_2_O)]_n_ with µ-1,1,3-azide (4-abaH = 4-aminobenzoic acid; Chen & Chen, 2002). The cis N—Cd—N bond angles are in the range of 74.11 (9) to 98.95 (8)°, deviating much from 90°.

A pair of azide ligands bridge the Cd^II^ atoms to form a [Cd_2_(N_3_)_2_] four-membered metallacycle. The neighboring metallacycles have a common Cd^II^ atom and form a one-dimensional inorganic zigzag chain [Cd(N_3_)_2_]_n_ (Fig. 2). The Cd1^···^Cd1# and Cd1^···^Cd1& separations *via* the EO-azide ligands are 3.6804 (7) and 3.7688 (7) Å, respectively. There is one symmetry independent bime ligand in (I). The bime ligands exhibits the anti-conformation with the torsion angle N1—C1—C2—N3 of 179.4 (2)°. The Cd^···^Cd distances between Cd^II^ atoms bridged *via* bime ligands are 11.5566 (15) Å.

The bime ligands attached to the [Cd(N_3_)_2_]_n_ chain point in four different directions (Fig. 3) binding to adjacent [Cd(N_3_)_2_ chain and generating a three-dimensional network (Fig. 4).

### Experimental

An aqueous (20 mL) solution of Cd(NO_3_)_2_^.^4H_2_O (0.50 mmol) and NaN_3_ (1.0 mmol) was added to one side of a "H-shape" tube, and a methanolic solution (20 mL) of bime (0.50 mmol) was added to the another side of the "H-shape" tube. Colourless crystal were obtained after about one month. Anal. Calcd. for C_8_H_10_CdN_10_: C, 26.79; H, 2.81; N, 39.06%. Found: C, 26.68; H, 2.72; N, 38.98%.

### Refinement

H atoms were placed in idealized positions and refined as riding, with C—H distances of 0.99Å (ethyl) and 0.95Å (imidazole) with *U*_iso_(H) = 1.2*U*_eq_(C).

### Crystal data

**Table:**

[Cd(N_3_)_2_(C_8_H_10_N_4_)]	*F*(000) = 704
*M**_r_* = 358.66	*D*_x_ = 1.871 Mg m^−^^3^
Monoclinic, *P*2_1_/*c*	Mo *K*α radiation, λ = 0.71070 Å
Hall symbol: -P 2ybc	Cell parameters from 4950 reflections
*a* = 6.4565 (14) Å	θ = 3.2–25.4°
*b* = 18.874 (4) Å	µ = 1.72 mm^−^^1^
*c* = 10.449 (2) Å	*T* = 153 K
β = 90.485 (5)°	Block, colourless
*V* = 1273.2 (5) Å^3^	0.36 × 0.17 × 0.15 mm
*Z* = 4	

### Data collection

**Table:**

Rigaku Mercury CCD diffractometer	2324 independent reflections
Radiation source: fine-focus sealed tube	2190 reflections with *I* > 2σ(*I*)
Graphite monochromator	*R*_int_ = 0.029
ω scans	θ_max_ = 25.3°, θ_min_ = 3.2°
Absorption correction: multi-scan (REQAB; Jacobson, 1998)	*h* = −7→7
*T*_min_ = 0.576, *T*_max_ = 0.783	*k* = −22→21
12260 measured reflections	*l* = −12→12

### Refinement

**Table:**

Refinement on *F*^2^	Primary atom site location: structure-invariant direct methods
Least-squares matrix: full	Secondary atom site location: difference Fourier map
*R*[*F*^2^ > 2σ(*F*^2^)] = 0.023	Hydrogen site location: inferred from neighbouring sites
*wR*(*F*^2^) = 0.056	H-atom parameters constrained
*S* = 1.09	*w* = 1/[σ^2^(*F*_o_^2^) + (0.0271*P*)^2^ + 1.003*P*] where *P* = (*F*_o_^2^ + 2*F*_c_^2^)/3
2324 reflections	(Δ/σ)_max_ < 0.001
172 parameters	Δρ_max_ = 0.68 e Å^−^^3^
0 restraints	Δρ_min_ = −0.39 e Å^−^^3^

### Special details

**Table:**

Geometry. All e.s.d.'s (except the e.s.d. in the dihedral angle between two l.s. planes) are estimated using the full covariance matrix. The cell e.s.d.'s are taken into account individually in the estimation of e.s.d.'s in distances, angles and torsion angles; correlations between e.s.d.'s in cell parameters are only used when they are defined by crystal symmetry. An approximate (isotropic) treatment of cell e.s.d.'s is used for estimating e.s.d.'s involving l.s. planes.
Refinement. Refinement of *F*^2^ against ALL reflections. The weighted *R*-factor *wR* and goodness of fit *S* are based on *F*^2^, conventional *R*-factors *R* are based on *F*, with *F* set to zero for negative *F*^2^. The threshold expression of *F*^2^ > σ(*F*^2^) is used only for calculating *R*-factors(gt) *etc*. and is not relevant to the choice of reflections for refinement. *R*-factors based on *F*^2^ are statistically about twice as large as those based on *F*, and *R*- factors based on ALL data will be even larger.

### Fractional atomic coordinates and isotropic or equivalent isotropic displacement parameters (Å^2^)

**Table:**

	*x*	*y*	*z*	*U*_iso_*/*U*_eq_	
Cd1	0.25318 (3)	0.538064 (9)	0.443664 (17)	0.01313 (8)	
N1	0.6247 (3)	0.60924 (12)	0.1163 (2)	0.0180 (5)	
N2	0.4047 (3)	0.56039 (12)	0.2487 (2)	0.0173 (5)	
N3	0.9231 (4)	0.75635 (12)	−0.0341 (2)	0.0214 (5)	
N4	1.1350 (4)	0.84573 (12)	−0.0646 (2)	0.0203 (5)	
N5	0.5555 (3)	0.57080 (12)	0.5537 (2)	0.0174 (5)	
N6	0.5556 (4)	0.61004 (12)	0.6435 (2)	0.0210 (5)	
N7	0.5618 (5)	0.64938 (15)	0.7285 (3)	0.0419 (8)	
N8	−0.0515 (3)	0.48306 (13)	0.3709 (2)	0.0194 (5)	
N9	−0.0800 (3)	0.46469 (11)	0.2657 (2)	0.0163 (5)	
N10	−0.1107 (4)	0.44775 (15)	0.1588 (3)	0.0344 (7)	
C1	0.8087 (4)	0.64601 (16)	0.0693 (3)	0.0228 (6)	
H1A	0.8769	0.6170	0.0030	0.027*	
H1B	0.9085	0.6534	0.1405	0.027*	
C2	0.7448 (4)	0.71673 (15)	0.0134 (3)	0.0244 (6)	
H2A	0.6452	0.7088	−0.0578	0.029*	
H2B	0.6742	0.7450	0.0798	0.029*	
C3	0.5867 (4)	0.59166 (14)	0.2384 (3)	0.0182 (6)	
H3A	0.6786	0.6005	0.3081	0.022*	
C4	0.3236 (4)	0.55828 (15)	0.1267 (3)	0.0212 (6)	
H4A	0.1927	0.5388	0.1039	0.025*	
C5	0.4581 (4)	0.58817 (15)	0.0439 (3)	0.0223 (6)	
H5A	0.4405	0.5934	−0.0460	0.027*	
C6	0.9741 (4)	0.82267 (14)	−0.0004 (3)	0.0202 (6)	
H6A	0.9028	0.8497	0.0621	0.024*	
C7	1.1899 (5)	0.79080 (16)	−0.1438 (3)	0.0322 (8)	
H7A	1.3012	0.7917	−0.2027	0.039*	
C8	1.0618 (5)	0.73543 (17)	−0.1252 (3)	0.0350 (8)	
H8A	1.0667	0.6908	−0.1669	0.042*	

### Atomic displacement parameters (Å^2^)

**Table:**

	*U*^11^	*U*^22^	*U*^33^	*U*^12^	*U*^13^	*U*^23^
Cd1	0.01024 (12)	0.01476 (13)	0.01442 (12)	0.00040 (7)	0.00091 (8)	0.00122 (7)
N1	0.0179 (12)	0.0179 (12)	0.0184 (12)	−0.0024 (9)	0.0052 (10)	0.0028 (9)
N2	0.0180 (12)	0.0174 (11)	0.0164 (12)	0.0028 (10)	0.0023 (10)	0.0012 (10)
N3	0.0223 (13)	0.0169 (12)	0.0251 (13)	−0.0057 (10)	0.0059 (11)	−0.0022 (10)
N4	0.0184 (12)	0.0173 (12)	0.0252 (13)	−0.0019 (10)	0.0036 (10)	−0.0013 (10)
N5	0.0156 (12)	0.0167 (12)	0.0199 (12)	0.0020 (9)	−0.0006 (10)	−0.0020 (10)
N6	0.0192 (13)	0.0205 (13)	0.0233 (14)	−0.0054 (10)	0.0037 (10)	0.0033 (11)
N7	0.063 (2)	0.0351 (17)	0.0281 (16)	−0.0174 (15)	0.0124 (14)	−0.0144 (13)
N8	0.0136 (12)	0.0269 (13)	0.0178 (13)	−0.0039 (10)	0.0006 (10)	0.0029 (11)
N9	0.0108 (11)	0.0136 (12)	0.0246 (15)	−0.0019 (8)	0.0031 (10)	0.0020 (10)
N10	0.0336 (16)	0.0459 (17)	0.0238 (16)	−0.0125 (13)	0.0015 (12)	−0.0103 (13)
C1	0.0167 (14)	0.0288 (16)	0.0231 (15)	−0.0040 (12)	0.0042 (12)	0.0038 (12)
C2	0.0205 (15)	0.0215 (15)	0.0312 (16)	−0.0055 (12)	0.0040 (13)	0.0020 (13)
C3	0.0161 (14)	0.0204 (14)	0.0182 (14)	0.0016 (11)	−0.0005 (11)	0.0010 (11)
C4	0.0203 (15)	0.0209 (14)	0.0222 (15)	−0.0042 (12)	−0.0011 (12)	−0.0031 (12)
C5	0.0252 (16)	0.0241 (15)	0.0177 (14)	−0.0061 (12)	0.0001 (12)	−0.0012 (12)
C6	0.0199 (15)	0.0161 (14)	0.0247 (15)	−0.0019 (11)	0.0056 (12)	−0.0025 (11)
C7	0.0392 (19)	0.0233 (16)	0.0345 (18)	−0.0055 (14)	0.0204 (15)	−0.0058 (13)
C8	0.049 (2)	0.0218 (16)	0.0344 (18)	−0.0084 (15)	0.0219 (16)	−0.0103 (14)

### Geometric parameters (Å, º)

**Table:**

Cd1—N2	2.306 (2)	N5—Cd1^iii^	2.397 (2)
Cd1—N4^i^	2.324 (2)	N6—N7	1.158 (3)
Cd1—N5	2.340 (2)	N8—N9	1.166 (3)
Cd1—N8	2.345 (2)	N8—Cd1^ii^	2.377 (2)
Cd1—N8^ii^	2.377 (2)	N9—N10	1.177 (4)
Cd1—N5^iii^	2.397 (2)	C1—C2	1.513 (4)
N1—C3	1.343 (3)	C1—H1A	0.9900
N1—C5	1.369 (4)	C1—H1B	0.9900
N1—C1	1.464 (4)	C2—H2A	0.9900
N2—C3	1.320 (4)	C2—H2B	0.9900
N2—C4	1.375 (4)	C3—H3A	0.9500
N3—C6	1.341 (3)	C4—C5	1.355 (4)
N3—C8	1.370 (4)	C4—H4A	0.9500
N3—C2	1.463 (4)	C5—H5A	0.9500
N4—C6	1.316 (4)	C6—H6A	0.9500
N4—C7	1.375 (4)	C7—C8	1.348 (4)
N4—Cd1^iv^	2.324 (2)	C7—H7A	0.9500
N5—N6	1.195 (3)	C8—H8A	0.9500
			
N2—Cd1—N4^i^	86.36 (8)	Cd1—N8—Cd1^ii^	105.89 (9)
N2—Cd1—N5	91.57 (8)	N8—N9—N10	178.4 (3)
N4^i^—Cd1—N5	92.36 (8)	N1—C1—C2	109.1 (2)
N2—Cd1—N8	98.95 (8)	N1—C1—H1A	109.9
N4^i^—Cd1—N8	97.56 (8)	C2—C1—H1A	109.9
N5—Cd1—N8	165.94 (8)	N1—C1—H1B	109.9
N2—Cd1—N8^ii^	171.89 (8)	C2—C1—H1B	109.9
N4^i^—Cd1—N8^ii^	90.38 (8)	H1A—C1—H1B	108.3
N5—Cd1—N8^ii^	95.98 (8)	N3—C2—C1	111.7 (2)
N8—Cd1—N8^ii^	74.11 (9)	N3—C2—H2A	109.3
N2—Cd1—N5^iii^	86.80 (8)	C1—C2—H2A	109.3
N4^i^—Cd1—N5^iii^	168.07 (8)	N3—C2—H2B	109.3
N5—Cd1—N5^iii^	78.07 (8)	C1—C2—H2B	109.3
N8—Cd1—N5^iii^	93.15 (8)	H2A—C2—H2B	107.9
N8^ii^—Cd1—N5^iii^	97.62 (8)	N2—C3—N1	111.0 (2)
C3—N1—C5	107.7 (2)	N2—C3—H3A	124.5
C3—N1—C1	126.2 (2)	N1—C3—H3A	124.5
C5—N1—C1	126.1 (2)	C5—C4—N2	109.8 (2)
C3—N2—C4	105.6 (2)	C5—C4—H4A	125.1
C3—N2—Cd1	122.58 (18)	N2—C4—H4A	125.1
C4—N2—Cd1	130.74 (18)	C4—C5—N1	105.8 (2)
C6—N3—C8	106.9 (2)	C4—C5—H5A	127.1
C6—N3—C2	125.5 (2)	N1—C5—H5A	127.1
C8—N3—C2	127.5 (2)	N4—C6—N3	111.6 (2)
C6—N4—C7	105.4 (2)	N4—C6—H6A	124.2
C6—N4—Cd1^iv^	123.63 (18)	N3—C6—H6A	124.2
C7—N4—Cd1^iv^	130.37 (19)	C8—C7—N4	109.6 (3)
N6—N5—Cd1	122.96 (18)	C8—C7—H7A	125.2
N6—N5—Cd1^iii^	121.70 (18)	N4—C7—H7A	125.2
Cd1—N5—Cd1^iii^	101.93 (8)	C7—C8—N3	106.4 (3)
N7—N6—N5	177.5 (3)	C7—C8—H8A	126.8
N9—N8—Cd1	124.23 (19)	N3—C8—H8A	126.8
N9—N8—Cd1^ii^	129.46 (19)		
			
C3—N1—C1—C2	−116.5 (3)	C3—N1—C5—C4	0.1 (3)
C5—N1—C1—C2	61.8 (4)	C1—N1—C5—C4	−178.4 (3)
C6—N3—C2—C1	−125.8 (3)	C7—N4—C6—N3	−0.1 (3)
C8—N3—C2—C1	58.2 (4)	Cd1^iv^—N4—C6—N3	172.20 (18)
N1—C1—C2—N3	179.4 (2)	C8—N3—C6—N4	0.5 (3)
C4—N2—C3—N1	−0.1 (3)	C2—N3—C6—N4	−176.2 (3)
Cd1—N2—C3—N1	−169.59 (17)	C6—N4—C7—C8	−0.5 (4)
C5—N1—C3—N2	0.0 (3)	Cd1^iv^—N4—C7—C8	−172.0 (2)
C1—N1—C3—N2	178.6 (2)	N4—C7—C8—N3	0.8 (4)
C3—N2—C4—C5	0.2 (3)	C6—N3—C8—C7	−0.8 (4)
Cd1—N2—C4—C5	168.5 (2)	C2—N3—C8—C7	175.9 (3)
N2—C4—C5—N1	−0.2 (3)		

Symmetry codes: (i) *x*−1, −*y*+3/2, *z*+1/2; (ii) −*x*, −*y*+1, −*z*+1; (iii) −*x*+1, −*y*+1, −*z*+1; (iv) *x*+1, −*y*+3/2, *z*−1/2.

## References

[bb1] Batten, S. R. & Robson, R. (1998). *Angew. Chem. Int. Ed.* **37**, 1460–1494. 10.1002/(SICI)1521-3773(19980619)37:11<1460::AID-ANIE1460>3.0.CO;2-Z 29710936

[bb2] Blake, A. J., Champness, N. R., Hubberstey, P., Li, W. S., Schroder, M. & Withersby, M. A. (1999). *Coord. Chem. Rev.* **183**, 117–138.

[bb3] Chen, H. J. & Chen, X. M. (2002). *Inorg. Chim. Acta*, **329**, 13–21.

[bb4] Hoskins, B. F., Robson, R. & Slizys, D. A. (1997*b*). *Angew. Chem. Int. Ed.* **36**, 2336–2338.

[bb5] Hoskins, B. F., Ronson, R. & Slizys, D. A. (1997*a*). *J. Am. Chem. Soc.* **119**, 2952–2953.

[bb6] Jacobson, R. (1998). *REQAB* Private communication to Rigaku Corporation, Tokyo, Japan.

[bb7] Kitagawa, S., Kitaura, R. & Noro, S. I. (2004). *Angew. Chem. Int. Ed.* **43**, 2334–2375. 10.1002/anie.200300610 15114565

[bb8] Leibeling, G., Demeshko, S., Bauer-Siebenlist, B., Meyer, F. & Pritzkow, H. (2004). *Eur. J. Inorg. Chem.* pp. 2413–2420.

[bb9] Mautner, F. A., Abu-Youssef, M. A. M. & Goher, M. A. S. (1997). *Polyhedron*, **16**, 235–242.

[bb10] Ribas, J., Escuer, A., Monfort, M., Vicente, R., Cortes, R., Lezama, L. & Rojo, T. (1999). *Coord. Chem. Rev.* **193–195**, 1027–1068.

[bb11] Rigaku (2000). *CrystalClear* Rigaku Corporation, Tokyo, Japan.

[bb12] Sheldrick, G. M. (2008). *Acta Cryst.* A**64**, 112–122. 10.1107/S0108767307043930 18156677

[bb13] Zhang, Y. P., Wang, L. Y., Wang, S. W., Li, B. L. & Zhang, Y. (2008). *J. Chem. Crystallogr.* **38**, 81–84.

[bb14] Zhang, Y., Wang, Z.-H., Zhang, Y.-P. & Li, B.-L. (2005). *Acta Cryst.* E**61**, m2722–m2725.

[bb15] Zhu, X., Zhao, J. W., Li, B. L., Song, Y., Zhang, Y. M. & Zhang, Y. (2010). *Inorg. Chem.* **49**, 1266–1270. 10.1021/ic902404b 20039702

